# Eyelid Skin Grafting in Young Patients with Facial Nerve Palsy

**DOI:** 10.3390/jcm13072142

**Published:** 2024-04-08

**Authors:** Yinon Shapira, Katja Ullrich, Sundas Masqood, Linda Okafor, Raman Malhotra

**Affiliations:** 1 Department of Ophthalmology, Carmel Medical Center, Haifa 3436212, Israel; 2 Department of Ophthalmology, Queen Elizabeth Hospital, Adelaide, SA 5011, Australia; 3 Corneoplastic Unit, Queen Victoria Hospital NHS Trust, East Grinstead RH19 3DZ, UK; sundas.maqsood1@nhs.net (S.M.);

**Keywords:** facial nerve palsy, skin graft, young patients, lagophthalmos, eyelid retraction, skin contraction, rehabilitation surgery

## Abstract

**Background**: The aim of this study is to report outcomes of eyelid full-thickness skin grafting augmentation in facial nerve palsy (FNP) patients younger than 50 years of age. **Methods**: In a retrospective, consecutive case series, nine eyelid skin grafts performed on eight FNP patients with skin contraction (five females; median age 42 years [range, 17–47]) are presented. In most cases, upper eyelid skin grafting was combined with levator recession and anterior lamellar repositioning. Lower eyelid skin grafting was combined with lower retractors recession in all cases. Functional and cosmetic outcomes were measured preoperatively and at early (1–3 months), intermediate (3–6 months), and late (≥12 months) follow-up. **Results**: The corneal score improved at early follow-up (*p* = 0.03) and remained improved at late follow-up (*p* = 0.042). The gentle closure lagophthalmos was improved at late follow-up (*p* = 0.042). (*p* = 0.048). The grades of graft color, edge/skin interface, and size improved at 3–6 months post-grafting and remained improved at late follow-up (*p* < 0.05). Over the follow-up, four patients (50%) were recommended to have further surgical procedures. **Conclusions**: The preliminary results from this small cohort suggest that eyelid skin grafting is a viable option for young patients prioritizing cosmesis. This technique warrants consideration for its functional benefits.

## 1. Introduction

Surgical options for periocular facial nerve palsy (FNP) rehabilitation include recession of retractors [1,2], eyelid loading [3], horizontal tightening [4], canthal plication [5,6], forehead, brow, and mid-face elevation [7,8], as well as volume restoration [9]. Nevertheless, eyelid position symmetry and adequate closure are often short-lived or insufficient, necessitating repeat procedures.

One of the potential reasons for early failure and poor outcomes may be soft tissue and skin contraction with subsequent eyelid retraction. Periocular skin contraction occurs in up to 71% of patients with FNP [10]. Using periocular full-thickness skin grafts may be a beneficial adjunct to other oculoplastic procedures for functional and cosmetic rehabilitation in patients with FNP, where skin contraction exists [11]. However, this has been reported in older adults (mean age 68), and clinicians may feel reluctant to employ periocular skin grafting in younger patients. To our knowledge, no previous reports assessed the outcomes of eyelid skin grafting in the rehabilitation of young patients with FNP. This series aims to report the functional and cosmetic outcomes of eyelid full-thickness skin grafts in the surgical rehabilitation of adults under the age of 50 years with FNP.

## 2. Methods

This was a single-center, retrospective, observational, consecutive case series. The medical charts of all FNP patients under 50 years of age who underwent periocular skin grafting at the Corneoplastic Unit of the Queen Victoria Hospital were reviewed. Past medical history, demographics, physical examination, surgical intervention, surgical outcomes, and illustrative preoperative and postoperative photography were retrieved from the medical records. The study was approved by the local institutional review board of Queen Victoria Hospital and was conducted according to the tenets of the Declaration of Helsinki. All patients gave written informed consent for all surgical procedures and the publication of photographs.

### 2.1. Patient Selection

Eyelid full-thickness skin grafting was performed after fulfillment of the following criteria:

1. Lagophthalmos and significant skin contraction were present. 2. Objective evidence for skin contraction included a reduced upper eyelid margin to brow distance (LMBD) of greater than 5 mm compared with the contralateral upper eyelid or an LMBD <25 mm [10]. 3. Skin grafting for the lower eyelids was determined based on the clinical evaluation, much like it is decided upon in cases where it is needed to correct cicatricial ectropion [12], that is, if lower lid retraction was noted with an inferior scleral show that was not deemed amenable to eyelid tightening alone (unlike paralytic retraction).

### 2.2. Outcome Measures

The functional outcome measures included corneal appearance (i.e., staining) graded according to a previously published grading scale [13,14], and lagophthalmos on blink, gentle, and forced closures as indicators of dynamic function. Based on standardized photographs, marginal reflex distance (MRD1 and MRD2) was calculated using a formula derived from Hashemi et al. [15]. In cases of upper eyelid grafting, pre- and postoperative LMBD measures are reported based on physical examination.

The cosmetic outcomes evaluation was conducted by two unbiased independent assessors who graded standardized photographs based on a previously published, mutually agreed grading scale [11]. The assessment addresses graft color, size, interface, pretarsal show symmetry, surface contour, and eyelid margin contour [11].

### 2.3. Surgical Technique

The surgical technique utilized for eyelid full-thickness skin grafting has been described previously [11]. Briefly, for upper eyelid grafts, a supraciliary skin incision is made across the eyelid with the eyelid on a downward stretch. A skin flap is raised superiorly between the skin and the orbicularis muscle, leaving an area of bare orbicularis. If recession of the levator is planned, then the septum is released for exposure of the levator aponeurosis. For lower eyelid grafts, a subciliary skin incision is made across the eyelid with the eyelid on an upward stretch. A horizontal stepped incision is made through the preseptal orbicularis, leaving the pretarsal orbicularis intact. The orbital septum is then released from canthus to canthus. If recession of the lower eyelid retractors is planned, then the retractors are exposed and recessed.

An adequately sized area of skin is marked and harvested from the preferred donor site, including postauricular or preauricular, supraclavicular, and suprabrow (in cases in which a direct brow lift was performed, and only in males). The full-thickness skin graft was defatted, placed into the desired area, and sutured using quilting and continuous 6.0 Monocryl (Ethicon, Raritan, NJ, USA) sutures. The harvest site was closed using a 5–0 Monocryl (Ethicon) continuous interlocking suture with or without deep interrupted sutures, as needed.

## 3. Results

Nine full-thickness skin grafts (five upper eyelids, four lower eyelids) were performed on eight FNP younger than 50 years between February 2013 and May 2019. Five of the patients were females, and the median age was 42 years (range, 17–47; three patients were under 40 years of age). The median postoperative follow-up time was 21 months (range, 12–54.5).

Table 1 presents the consecutive patient demographics, characteristics, donor sites, and procedures. All patients but one (case 2, aged 17 years) had undergone prior eyelid procedures. These included retractor recession of either the upper or lower eyelids, horizontal lower eyelid tightening, posterior limb medial canthal tendon (MCT) plication and suture sling, gray line split and anterior lamellar repositioning of either the upper or lower eyelids, autologous fat grafting, and one case of frontalis suspension ptosis correction. Seven of the eight patients underwent prior upper eyelid loading with gold weights or platinum chains (PC), some of which subsequently had either gold weight replacement with PC or removal of the weight. Consequently, at the time of the eyelid skin-grafting procedure, three out of eight patients had a PC, and two had a gold weight in situ.

Upper eyelid full-thickness skin grafting was combined with levator recession and anterior lamellar repositioning in the majority of cases. Concomitant lower eyelid retractors recession and lateral canthal suspension with/without MCT plication was carried out in two of the upper eyelid skin grafts cases, and in one case, a PC was removed (Table 1). Lower eyelid full-thickness skin grafting was combined with lower retractors recession in all cases.

Throughout the postoperative follow-up, no patient developed graft failure, hypertrophy, or hematoma. However, one patient (case 3, aged 44) developed upper eyelid skin graft contraction at six months and requested further skin grafting five years after the original operation (they are currently awaiting the procedure). One patient (case 2, age 17 years) needed surgical revision involving thinning of upper eyelid graft, with concomitant levator advancement, MCT plication, and fat grafting at ten months, postoperatively. This patient underwent a repeat levator advancement almost three years following this surgery combined with a gray line split and anterior lamellar repositioning. One patient (case 1, age 43) who underwent staged upper and then lower eyelids full-thickness skin graft needed medial tarsorrhaphy at 11 months postoperatively due to persistent lagophthalmos and corneal epitheliopathy. One patient (case 8, aged 36) underwent bilateral periocular autologous fat grafting at six months postoperatively due to bilateral lower eyelids retraction (associated with inferior volume deficit). In total, four patients (50%) were recommended or underwent further surgical procedures over their follow-up.

### 3.1. Clinical outcomes

Table 2 summarizes comparisons of mean functional outcomes (corneal appearance, MRD, LMBD, lagophthalmos) at early (1–3 months), intermediate (6–9 months), and late (≥12 months) postoperative review. At early follow-up, there was a significant improvement in the corneal staining mean score (from C score 2.2 ± 0.4 to 0.7 ± 1.0). A significant mean improvement was maintained at the late postoperative corneal assessment (C score 1.3 ± 0.5, Table 2).

In cases of upper skin grafting (n = 5), the mean MRD1 did not significantly change postoperatively (Table 2). Expressly, a reduction of 2 mm in MRD1 was noted in two cases, a reduction of 0.5 mm was noted in one case, a 0.5 mm increase was noted in one case, and a 1 mm increase (retraction) was noted in the case where the graft contracture occurred. In cases of lower eyelid grafting (n = 4), the mean MRD2 significantly improved (was reduced) at the late postoperative phase (from 7.3 ± 0.6 to 5.8 ± 0.8 mm, Table 2). Expressly, a 1.5 mm lower eyelid elevation was noted in three cases and 0.5 mm in one case.

The mean preoperative LMBD in the upper eyelid skin graft cases was reduced at 20.5 ± 4.2 mm. Postoperative LMBD clinical measurements were available for four out of five upper eyelid skin grafts. At intermediate follow-up, there was a significant mean increase (to 25.3 ± 8.5 mm) in the LMBD. At late-phase follow-up, the mean increase did not reach statistical significance (LMBD 23.3 ± 7.0, Table 2). Nevertheless, in three of the patients, LMBD increased by 5–8 mm late postoperatively, from 15–25 mm LMBD preoperatively to 20–30 mm. A relative decrease of 7 mm was noted in the one patient that experienced superior eyelid graft contraction.

There was a significant improvement in the mean gentle closure lagophthalmos at the late postoperative phase (Table 2, Supplement Figure S1). Namely, gentle closure improved by 2 mm in four cases, 3 mm in one, 5 mm in one, and did not change in two cases. In the case that experienced graft contraction, a 2 mm worsening of gentle closure lagophthalmos was noted.

In comparison to the baseline, the visual acuity improved on the final follow-up in four patients (from 6/12–6/60 to 6/9.5–6/38), did not change in one patient (6/9.5), and was worse in three patients (from 6/7.5–6/30 to 6/9.5–6/60).

### 3.2. Cosmetic Outcomes

**Figure 1:**
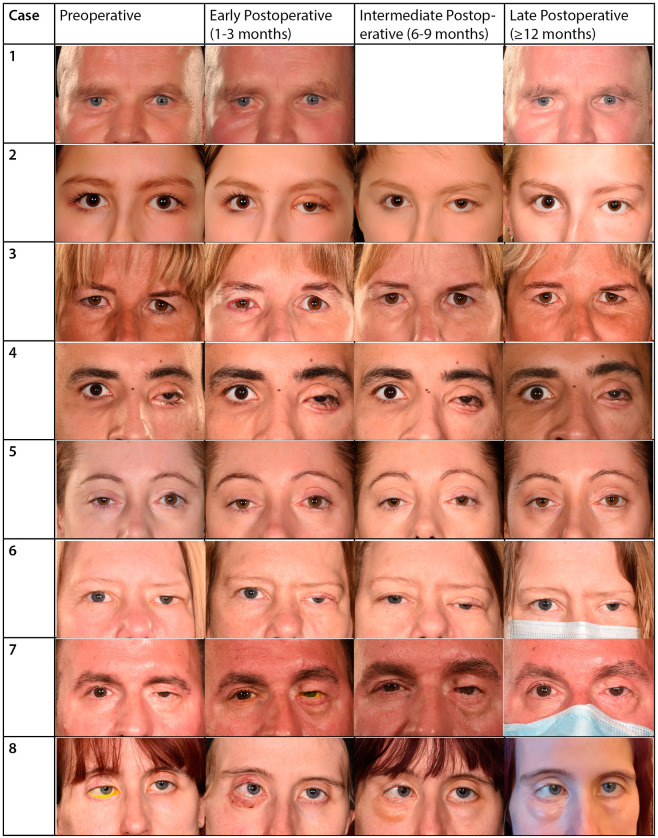
Comparing standardized photographs in eight consecutive young adults with facial nerve palsy (P1–P8) at pre-skin grafting and early, intermediate, and late postoperative follow-up. (P1) Preoperative—before right lower eyelid full-thickness skin graft. Postoperative—late postoperative result was taken 10 months following a medial tarsorrhaphy. (P2) Preoperative—before left upper full-thickness skin graft. Postoperative—the patient underwent thinning of the left upper graft, as well as levator advancement, MCT plication and fat grafting 10 months postoperatively. Almost three years later, the patient underwent a repeat levator advancement combined with a gray line split and anterior lamellar repositioning. The late postoperative photography was taken 11 months after the latter surgery. (P3) Preoperative—before right upper full-thickness skin graft. Postoperative—right upper eyelid graft contraction noted at the intermediate postoperative phase. The patient is awaiting repeat skin grafting. (P4) Preoperative—before left lower full-thickness skin graft. Postoperative—despite improvement in eyelid contraction, the skin graft cosmetic outcome was not satisfactory. (P5) Preoperative—before left upper full-thickness skin graft. Postoperative—there was improvement in eyelid contraction and closure, however the resultant graft contour was not ideal. (P6) Preoperative—before left upper full-thickness skin graft. Postoperative—improvement in eyelid closure, however the pretarsal show symmetry was not ideal. (P7) Preoperative—before left lower full-thickness skin graft combined with left brow lift. Postoperative—good graft and overall cosmetic outcome. (P8) Preoperative—before right lower full-thickness skin graft. Postoperative—the intermediate-phase photograph was taken 1 month following bilateral lower periocular fat grafting. The patient is awaiting revision of the fat grafting due to prominence.

Through a mutually agreed photographic grading scale [11], the two independent assessors graded cosmetic outcome measures (Table 3) for each postoperative period. Figure 1 illustrates the preoperative and postoperative periocular appearance of all included patients. One case (case 3, aged 44 years) was excluded from the photographic cosmetic assessment due to the significant contracture of the full-thickness skin graft, precluding grading of results (Figure 1).

The mean grades of graft color, edge/skin interface, and size significantly improved from the early to the intermediate postoperative period (Table 2). These parameters remained improved (or further improved) in the late postoperative phase (Table 2). The final grades for these parameters ranged from good to excellent in all patients except for case 4 (aged 29 years), in which they were good (color) and unsatisfactory (edge/skin interface and size).

The graft skin surface contour mean grade did not improve significantly in the intermediate postoperative phase; however, it significantly improved late postoperatively (Table 2). The final contour was graded good to excellent in all cases except for case 4 and case 5 (age 41 years), in which they were graded unsatisfactory (Figure 1).

The eyelid margin contour was not affected by the skin grafts in all cases but case 6 (aged 42, Figure 1). Specifically, in the early postoperative phase, the eyelid margin was graded as good to excellent in all cases except case 6 (unsatisfactory), and the grading did not change during the intermediate and late postoperative phases.

Of four upper eyelid full-thickness skin grafts included in the cosmetic outcomes analysis, all were graded unsatisfactory in pretarsal show/skin crease symmetry during the early postoperative phase. In the intermediate postoperative phase, two cases (case 1A, aged 43 years, and case 5, aged 41 years) improved to good-excellent grades, while cases 2 (aged 17 years) and 6 (aged 42 years) remained unsatisfactory (Figure 1). At the late postoperative phase, case 6 remained unsatisfactory, while case 2 improved, exhibiting good pretarsal show/skin crease symmetry after thinning of the graft was conducted.

## 4. Discussion

In facial nerve palsy, a subset of patients develops significant skin contraction. Local atrophy of muscle and soft tissue could result from disuse or diminished activity caused by the palsy. Furthermore, muscle-pump paralysis could also reduce the venous tone and raise the hydrostatic pressure within tissue. These may lead to fibrosis of the subcutis and trophic skin changes [10,16]. Traditional eyelid-tightening and eyelid-loading principles are often doomed to fail in these patients unless the tissue is augmented with skin [17].

These patients should not be encouraged to accept eyelid retraction, scleral show, and lagophthalmos as an inherent consequence of their FNP. Our experience shows that these cases could be improved by addressing skin contraction as a culprit [11]. The current report suggests that the option of skin grafting should not be ignored in younger patients where cosmesis may be considered a priority. With careful counseling, these patients can expect a natural skin appearance after approximately 6–9 months. This study helps provide evidence to support this when counseling such patients.

Our data also confirm our experience that these patients often need multiple procedures in the long term to address an ongoing contraction process. Consequently, follow-up for these individuals should be continued beyond a year. Specifically, three out of eight patients (37.5%) underwent further surgery, one for recurrent eyelid retraction, one for graft thinning, and one for persistent lagophthalmos. Two of these patients were under the age of 40, constituting two-thirds of the patients defined as young adults in this series. While the numbers are too small for a firm comparison, numerically, the patients in the young adult age group had higher re-operation rates (compared to the patients aged 41–47 years old).

Based on these results, it is important for surgeons to recognize that within this specific group of patients with severe facial palsy, contracture and retraction may continue to develop even after surgical rehabilitation that includes skin grafting. This understanding should inform the management of patient expectations, emphasizing the need for long-term follow-up. Although repeat surgeries might be necessary over time, traditional tarsorrhaphy—often considered cosmetically and functionally undesirable—should be viewed as a last resort.

In a previous study, we have shown that in FNP patients with Bell’s palsy as the underlying etiology, 62% (20/32) had evidence of upper-eyelid skin contracture in comparison with 87% (20/23) of patients with facial nerve resection in the context of surgery for acoustic schwannoma or parotid tumor, or trauma [10]. In the current series, none of these young patients had Bell’s palsy as the underlying etiology, and in fact, six out of eight cases belonged to the latter category. This supports our clinical experience that patients with more severe FNP often show more significant tissue contracture, perhaps due to a more profound lack of muscle activity [10,18].

Traditional classifications of FNP categorize its severity based on the degree of nerve injury (ranging from conduction block to complete nerve transection), with corresponding decreasing volitional electromyography (EMG) and increasing pathologic spontaneous EMG activity. The chance of recovery is also inversely related to the degree of nerve injury [19,20]. The degree of FNP was also the main predicting factor of health-related quality of life in such patients [21]. The current series of patients represents a high severity on the FNP spectrum, evidenced by the significant, continuing periocular tissue contracture and resultant eyelid retraction. Nevertheless, using skin graft augmentation in combination with other procedures (often necessitating more than one surgical intervention), these young patients managed to improve their ocular surface and dynamic function while maintaining an acceptable aesthetic appearance. Only one patient required resorting to (partial) tarsorrhaphy at late follow-up. It should be acknowledged that separating the functional effect of skin grafting alone from concurrent or even successive eyelid surgeries would be impossible. Consequently, the functional results cannot be attributed to the skin grafting alone. 

Another helpful observation based on these results is that it is safe to oversize the skin graft in young patients, expecting approximately 50% graft contracture (a qualitative observation). While only one patient required a repeat graft due to contraction, perhaps larger grafts could have further improved the outcomes in some of the other cases as well, possibly avoiding or deferring the need for subsequent surgery. Our observation is supported by a previous investigation into patterns of facial full-thickness skin graft contraction, reporting that eyelid skin grafts tend to contract by a mean of 49% (range −17% to −82%) in comparison to a mean of 27% (range +5% to −54%) in scalp or temple recipient sites. No correlation was found between the patient’s age and the degree of contraction [22]. Thus, when electing to employ skin grafting in young patients with FNP, graft oversizing considerations should be equivalent to those in older adults. The concern for cosmesis in young patients is a pitfall that should be evaded, as a large skin graft will contract and blend in, while the effectiveness of a cautiously sized graft is uncertain. Furthermore, postoperative graft thinning, utilized in one of our cases, remains an option for improving cosmesis while maintaining effectiveness, if needed.

Notably, regarding the cosmetic outcomes, young patients should be a more challenging group to achieve a suitable skin match with skin grafting and hence to please, because their recipient skin is inherently smoother. Therefore, the graft has to look excellent in order to be acceptable. The fact that in most cases, the cosmetic grading was high and similar to the reported outcomes in older patients (older than 60 years) [11] suggests that good aesthetic outcomes could be expected, even in younger patients. Furthermore, the final cosmetic scores were comparable between the three young adults (<40 years) and the six patients aged 41–47 years.

Finally, a major limitation of the current study is the small sample size. This is a highly specific group of patients—young patients with severe facial nerve palsy with significant skin contracture meriting skin grafting as augmentation, in all cases performed in concurrence with other eyelid procedures (e.g., lower eyelid tightening, retractors recession, upper eyelid implantation of weights or levator recession, etc.). Employing the skin grafting option in young FNP patients was practiced judiciously only in cases where it was determined to be crucial for augmentation during other traditional procedures. As a result, only a handful of such procedures have been performed in one of the largest plastic surgery referral centers in the UK, specializing in facial palsy rehabilitation procedures. Our data are particularly limited in patients under 40 years old, as this series includes only three such individuals. Despite this limitation, we believe these preliminary results provide some evidence, for the first time, that eyelid skin augmentation may have a place in the surgical rehabilitation of these rare cases. 

In conclusion, to the best of our knowledge, this experience of eyelid skin grafting within a young patient population with facial nerve palsy is the first to be reported to date. Thus, within the limitation of a small sample size, this audit determined whether skin grafting could be recommended to younger people and is useful to consider in their consenting. Taken together, this may be considered a pilot study yielding results that provide preliminary evidence of the feasibility of this approach in younger FNP patients. Further, larger studies are warranted to validate these preliminary findings. These future studies should also assess patient satisfaction, quality of life parameters, and blink dynamics [23].

## Figures and Tables

**Table 1:** Eyelid full-thickness skin grafting in young facial nerve palsy patients with skin contraction and eyelid retraction.

Case	Age	Gender	Etiology of FNP	Preceding Procedures	Eyelid	Donor Site	Concomitant Procedure	Final Follow Up (Months)
1A	43	M	Congenital	Upper gold weight, Replace gold weight with PC, Levator recession, Lower MCT plication + canthal repositioning, AFG cheek	Right Upper	Supraclavicular	Levator recession	33
1B	44				Right Lower	Supraclavicular	Lower retractors recession	21
2	17	F	Traumatic		Left Upper	Preauricular	Levator recession, Lower retractors recession	54.5
3	44	F	Congenital	Upper gold weight, Replace gold weight with PC, Removal PC	Right Upper	Postauricular	Upper gray line split + anterior lamella repositioning, Levator recession, Lower retractors recession + MCT plication + suture sling	22.5
4	29	M	Traumatic	Upper gold weight, Corneal neurotisation	Left Lower	Postauricular	Scar release + Lower retractors recession	15.5
5	41	F	Iatrogenic (excision meningioma)	Upper gold weight, removal of gold weight	Left Upper	Postauricular	Upper anterior lamella repositioning	12
6	42	F	Iatrogenic (chostochondral grafting)	Upper gold weight, gold weight repositioning, lower gray line split + anterior lamella repositioning + retractors recession	Left Upper	Supraclavicular	Upper gray line split + anterior lamellar repositioning, lower retractors recession + canthoplasty	19
7	47	M	Traumatic	Upper PC, Upper anterior lamella repositioning, lower MCT plication + suture sling + punctal cautery + retractors recession, Upper facia latta sling (frontalis suspension)	Left Lower	Suprabrow	Direct brow lift + lower retractors recession	27
8	36	F	Congenital	Upper PC, lower MCT plication + suture sling + retractors recession	Right lower	Postauricular	Upper removal of PC, lower retractors recession	13

FNP—facial nerve palsy; PC—platinum chain (weight); MCT—medial canthal tendon; AFG—autologous fat graft.

**Table 2:** Functional outcomes after eyelid full-thickness skin grafting in young facial nerve palsy patients.

Functional Outcomes	Cases	Preoperative Mean (SD)	Postoperative Mean (SD)					
			1–3 months	*p* ^1^	6–9 months	*p* ^1^	≥12 months	*p* ^1^
Corneal appearance	All	2.2 (0.4)	0.7 (1.0)	**0.030**	1.5 (0.8)	0.24	1.3 (0.5)	**0.042**
LMBD	Upper lid ^2^	20.4 (3.7)	25.3 (8.5)	0.28	25.0 (5.2)	**0.042**	23.1 (7.0)	0.47
MRD1	Upper lid ^2^	3.3 (1.9)	1.6 (1.5)	0.14	2.1 (1.3)	0.23	2.4 (1.1)	0.39
MRD2	Lower lid ^3^	7.3 (0.6)	6.0 (1.4)	0.063	5.2 (0.8)	**0.011**	5.8 (0.7)	**0.017**
Lagophthalmos blink	All	7.5 (1.5)	4.9 (1.6)	**0.005**	6.0 (2.0)	0.072	5.3 (2.8)	0.052
Lagophthalmos gentle	All	4.7 (2.0)	3.1 (2.4)	0.054	3.5 (2.6)	0.13	3.1 (3.1)	**0.048**
Lagophthalmos Forced	All	3.4 (2.4)	1.3 (1.6)	**0.011**	1.8 (2.6)	0.13	2.1 (3.9)	0.11

^1^ *p* value for dependent samples (paired) *t*-test comparison to preoperative value. ^2^ Assessed in upper eyelid grafting cases. ^3^ Assessed in lower eyelid grafting cases. LMBD lid margin to brow distance; MRD marginal reflex distance. Bold figures denote significant *p* values (<0.05).

**Table 3:** Cosmetic outcomes after eyelid full-thickness skin grafting in young facial nerve palsy patients.

Cosmetic Outcomes	Cases	Postoperative Grading Mean ^2^ (SD)				
		1–3 months	6–9 months	*p* ^1^	≥12 months	*p* ^1^
Graft color	All	1.8 (0.4)	0.8 (0.5)	**0.020**	0.6 (0.7)	**0.012**
Edge/interface	All	1.9 (0.6)	0.9 (0.7)	**0.032**	0.9 (0.6)	**0.013**
Skin surface contour	All	1.8 (0.5)	1.5 (0.5)	0.20	1.0 (0.7)	**0.042**
Size	All	1.5 (0.3)	0.8 (0.4)	**0.010**	0.8 (0.6)	**0.010**
Eyelid margin contour	All	1.0 (0.6)	0.8 (0.6)	0.17	0.9 (0.6)	0.17
Pretarsal show/skin crease symmetry	Upper lid ^3^	2.2 (0.3)	1.5 (0.9)	0.27	1.0 (1.0)	0.19

^1^ *p* value for dependent samples (paired) *t*-test comparison to early (1–3 months) postoperative value. ^2^ Photographic grading by two independent assessors (Range: 0—excellent to 3—unacceptable result). ^3^ Assessed in upper eyelid grafting cases. Bold figures denote significant *p* values (<0.05).

## Data Availability

Due to privacy issues only de-identified material may be made available upon request to interested researchers.

## Supplementary Materials

The following supporting information can be downloaded at: https://www.mdpi.com/article/10.3390/jcm13072142/s1, Supplement Figure S1: Standardized photographs with gentle eye closure in eight consecutive young adults with facial nerve palsy (P1–P8) at pre-skin grafting and late postoperative follow-up.
