# Supplementary material for: Eyelid Skin Grafting in Young Patients with Facial Nerve Palsy
Source: J Clin Med. 2024 Apr 8;13(7):2142. doi: 10.3390/jcm13072142 (PMC11012737; doi:10.3390/jcm13072142)
Supplement: Supplementary file 1 [file jcm-13-02142-s001.zip › jcm-2881468-supplementary.pdf]

Case	Preoperative	Late Postoperative
1	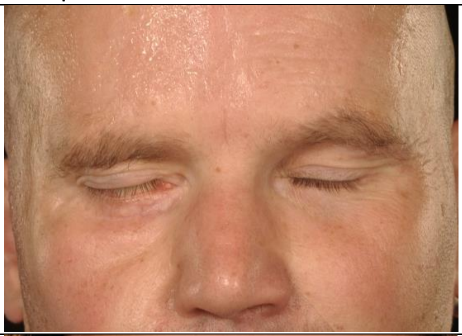	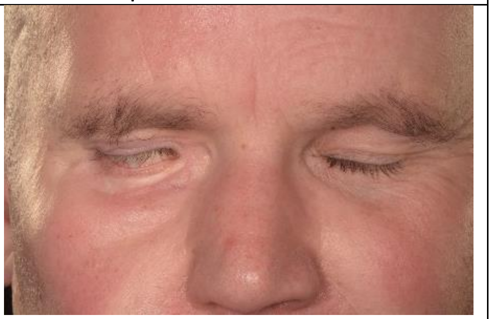
2	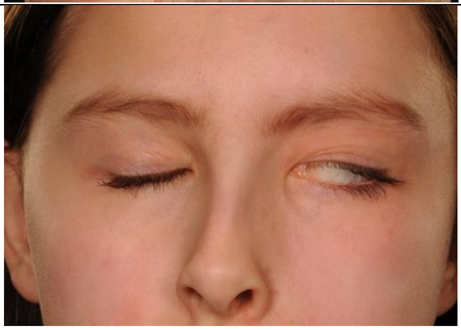	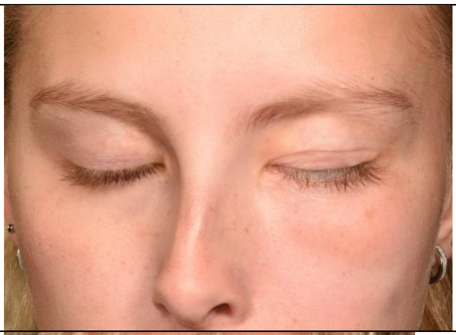
3	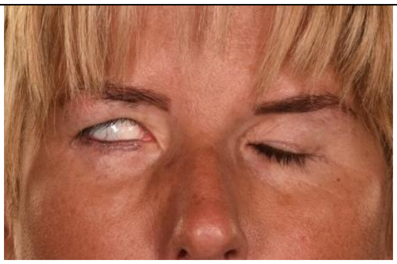	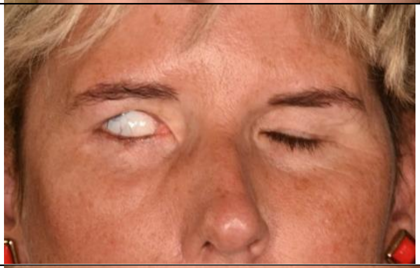
4	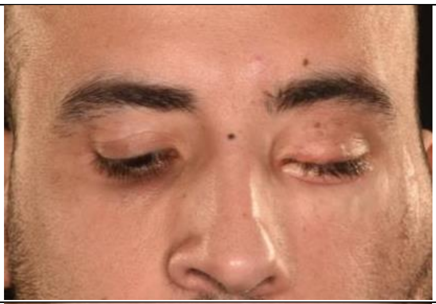	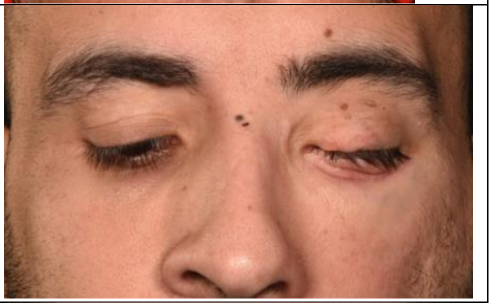
5	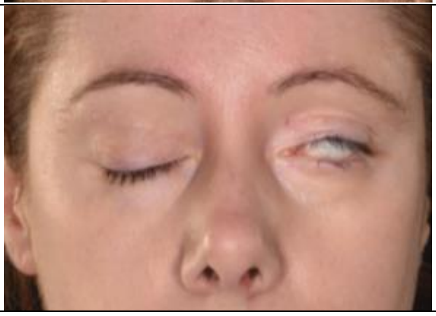	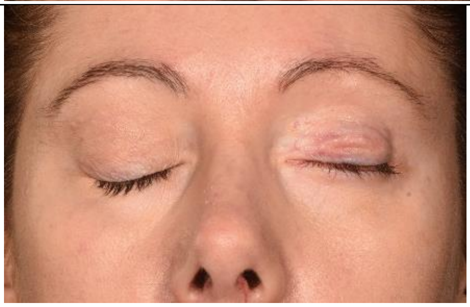

6	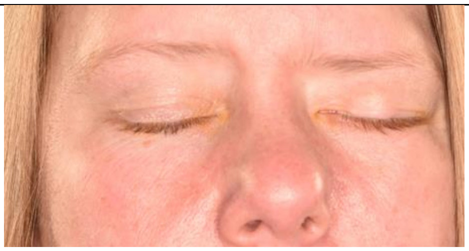	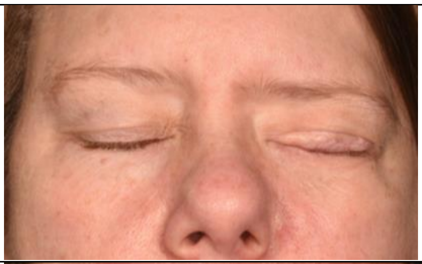
7	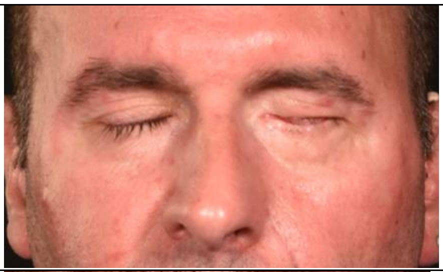	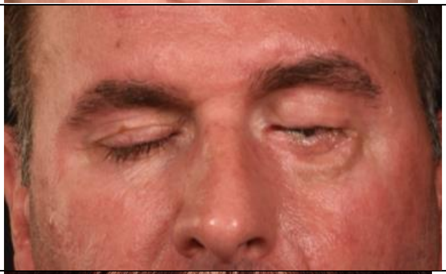
8	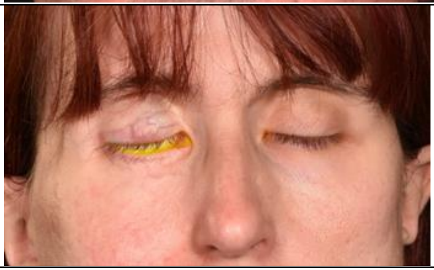	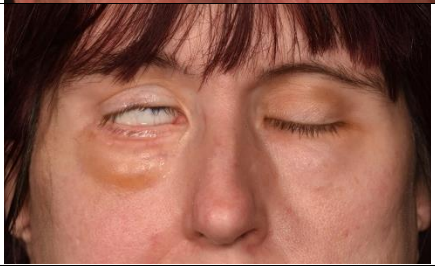

Supplement Figure S1:

Standardized photographs with gentle eye closure in eight consecutive young adults with facial nerve palsy (P1–P8) at pre-skin grafting and late postoperative follow-up.
